# CPA: a web-based platform for consensus pathway analysis and interactive visualization

**DOI:** 10.1093/nar/gkab421

**Published:** 2021-05-25

**Authors:** Hung Nguyen, Duc Tran, Jonathan M Galazka, Sylvain V Costes, Afshin Beheshti, Juli Petereit, Sorin Draghici, Tin Nguyen

**Affiliations:** University of Nevada Reno, Department of Computer Science and Engineering, Reno, NV 89557, USA; University of Nevada Reno, Department of Computer Science and Engineering, Reno, NV 89557, USA; NASA Ames Research Center, Space Biosciences Division, Moffett Field, CA 94035, USA; NASA Ames Research Center, Space Biosciences Division, Moffett Field, CA 94035, USA; KBR, NASA Ames Research Center, Space Biosciences Division, Moffett Field, CA 94035, USA; University of Nevada Reno, Nevada Bioinformatics Center, Reno, NV 89557, USA; Wayne State University, Department of Computer Science, Detroit, MI 48202, USA; University of Nevada Reno, Department of Computer Science and Engineering, Reno, NV 89557, USA

## Abstract

In molecular biology and genetics, there is a large gap between the ease of data collection and our ability to extract knowledge from these data. Contributing to this gap is the fact that living organisms are complex systems whose emerging phenotypes are the results of multiple complex interactions taking place on various pathways. This demands powerful yet user-friendly pathway analysis tools to translate the now abundant high-throughput data into a better understanding of the underlying biological phenomena. Here we introduce Consensus Pathway Analysis (CPA), a web-based platform that allows researchers to (i) perform pathway analysis using eight established methods (GSEA, GSA, FGSEA, PADOG, Impact Analysis, ORA/Webgestalt, KS-test, Wilcox-test), (ii) perform meta-analysis of multiple datasets, (iii) combine methods and datasets to accurately identify the impacted pathways underlying the studied condition and (iv) interactively explore impacted pathways, and browse relationships between pathways and genes. The platform supports three types of input: (i) a list of differentially expressed genes, (ii) genes and fold changes and (iii) an expression matrix. It also allows users to import data from NCBI GEO. The CPA platform currently supports the analysis of multiple organisms using KEGG and Gene Ontology, and it is freely available at http://cpa.tinnguyen-lab.com.

## Graphical Abstract

**Graphical Abstract:**
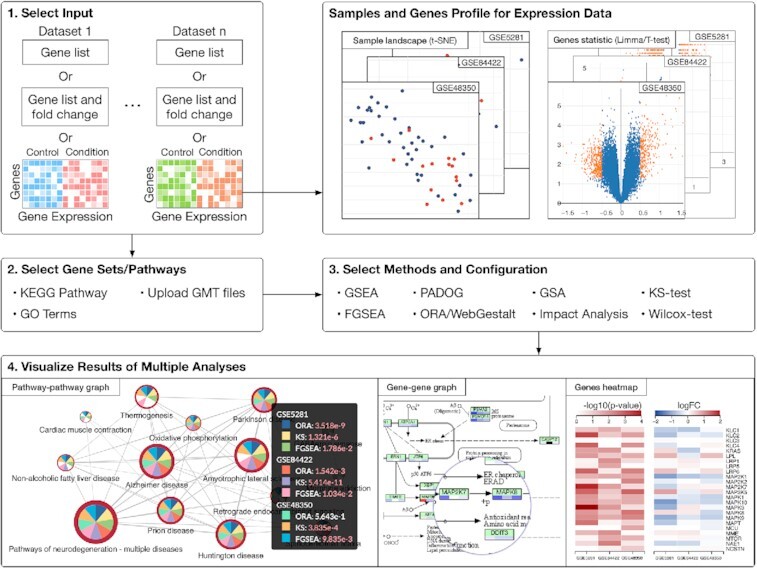
Pathway analysis and visualization using consensus pathway analysis (CPA). CPA allows users to compare, contrast and combine analysis results across different methods and experiments, and supports over 1000 organisms.

## INTRODUCTION

Advanced high-throughput and sequencing technologies have transformed biological research by allowing scientists to monitor changes in living organisms and biological systems. Regardless of the assay technology used, a comparative analysis experiment often yields a set of differentially expressed (DE) genes or gene products. Though important, these lists of DE genes fail to reveal the mechanisms underlying the studied condition. To translate the differential expression to biological knowledge, researchers have been developing various knowledge bases that map genes and their products to functional modules and biological processes. These include KEGG (1), Reactome (2), Wikipathways (3) and Gene Ontology (GO) (4). At the same time, pathway analysis methods have been developed to identify pathways that are impacted under certain conditions.

More than 70 pathway methods have been developed thus far (5,6). These methods can be categorized into three classes. The earliest approaches use Over-Representation Analysis (ORA) (7–12) that identify the pathways in which the DE genes are over- or under-represented. The drawbacks of ORA include: (i) it only considers the number of DE genes and completely ignores their expression changes and (ii) it assumes the genes are independent, which they are not. Functional Class Scoring (FCS) approaches (13–17) have been developed to address some of the issues raised by ORA approaches. The main improvement of FCS is based on the observation that small but coordinated changes in the expression of functionally related genes can have a significant impact on pathways. However, both ORA and FCS still ignore the direction and type of the signals between genes, the positions and roles of the genes on each pathway, as well as all the other information captured by the topology of the pathway. Topology-based (TB) approaches (18–25) which fully exploit all the knowledge about how genes interact as described by pathways, have been developed more recently. Recent reviews included 22 TB methods (6,26).

In spite of the availability of powerful pathway methods, understanding the phenomena that determine the measured changes is as challenging as ever, if not more so. First, the sheer number of methods makes it challenging for life scientists to choose the correct method for their data and purpose. In a recent publication (5), we have shown that all existing methods often provide biased results. No single method is consistently superior to others. Second, many of these methods are software packages that require users to go through the burden of installation and updating (some are not even executable anymore due to outdated dependencies). This hinders reproducibility and universal accessibility of analysis results. Finally, most tools do not offer interactive data visualizations that are important for users to deeply explore pathway connectivities and gene networks.

Recognizing these challenges, many web-based tools have been developed to assist researchers in their analysis. Tools such as EnrichNet (27), GENAVi (28), WebGestalt (29), WebGIVI (30), DAVID (31), INMEX (32), g:Profiler (33) and Enrichr (34) provide graphical user interfaces (GUIs) for users to input gene lists and perform enrichment analysis. Other tools such as KaPPA-View (35), 3Omics (36), PaintOmics (37), IMPaLA (38), and GeneTrail2 (39) visualize enrichment results of multi-omics data. These tools, however, have a number of limitations: (i) cannot combine, compare and contrast results of different methods, (ii) lack integrative capability across multiple datasets and (iii) unable to comprehensively visualize pathway connectivity, gene networks, and expression change all together.

Here, we introduce Consensus Pathway Analysis (CPA), a comprehensive web-based resource that allows users to compare and contrast analysis results across different methods and experiments. Specifically, CPA allows researchers to: i) perform pathway analysis using eight popular methods, GSEA (40), GSA (14), FGSEA (41,42), PADOG (16), Impact Analysis (19), ORA/WebGestalt (29,43), KS-test (44) and Wilcox-test (45), ii) perform meta-analysis of multiple datasets, iii) combine methods and datasets to find consensus results, and iv) interactively explore significantly impacted pathways across multiple analyses, and browse relationships between pathways and genes. CPA currently supports the analysis of more than 1000 organisms using KEGG and Gene Ontology databases.

## MATERIALS AND METHODS

The CPA website is a cloud-computing service for pathway analysis. It provides functions to manage users’ data, supports multiple analysis sessions and visualizes results. All computations are performed on the CPA server hosted by the University of Nevada, Reno (UNR). Inputs, parameter settings, and analysis results are saved onto the user account and can easily be loaded and updated. Users can also switch between analysis sessions, as well as browse and export results at any time.

**Figure 1.**
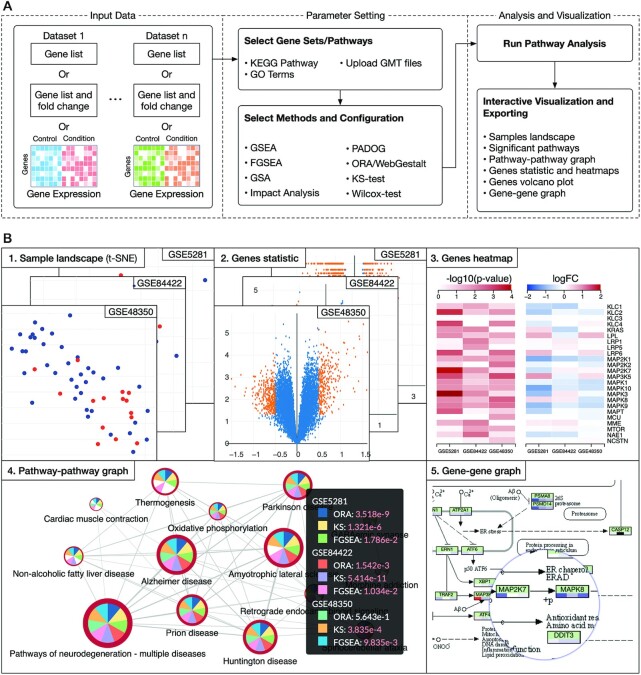
The overall workflow and data visualization using consensus pathway analysis (CPA). (**A**) The analysis pipeline consists of three modules: i) input data, ii) parameter setting, and iii) analysis and visualization. The input in one dataset can be a gene list, a gene list and their fold changes (FC) or an expression matrix. In one analysis session, CPA allows users to analyze multiple datasets using multiple pathway analysis methods. (**B**) Result visualization. Once the analysis is done, users can interactively explore and export the results. For example, they can export the samples landscape (B1), volcano plot (B2), and heatmaps showing *P*-values and log FC across all datasets (B3). At the pathway level, users can interactively visualize the pathway–pathway connectivity graph (B4) and KEGG pathways (B5). Users can see detailed analysis results and statistics by clicking on each node of graphs (B4). In this example, the analysis includes three datasets and three methods. Analysis results, plots and graphs can be exported as comma-separated values (.csv file) or publication-ready figures (.png, .svg, etc.).

Figure 1A shows the overall workflow of an analysis session using CPA while Figure 1B shows sample visualizations and analysis results. Overall, the analysis pipeline consists of three main modules: data input, parameter setting, and analysis and visualization. For input data, users can choose to input a gene list, a gene list and their fold changes, or a gene expression matrix from their local machine. The interface is designed so that users can flexibly analyze their own data. We also support a direct import from NCBI Gene Expression Omnibus (GEO) (46). This is especially helpful if users are interested in taking advantage of existing data on NCBI GEO. In parameter setting, users can choose the pathways of interest (GO/KEGG), analysis methods, and method parameters. Finally, in the analysis and visualization module, users can visualize and interactively explore and export analysis results. Figure 1B shows example visualizations and publication-ready figures generated by the platform. These include: sample landscape (using t-SNE), volcano plot, gene heatmap, pathway–pathway connectivity and gene networks. We will describe in details each of the three modules in the following sections.

### Input and data management

The CPA platform supports three different types of input including (i) a list of differentially expressed (DE) genes, (ii) genes and their fold changes and (iii) an expression matrix. The first two input types can be directly entered on the website or uploaded from users’ local machine as a .txt or .tsv file, in which each row represents a gene. For expression matrix input, a dataset can be represented by two .csv files (command-separated)—one for expression matrix and one for sample grouping. The sample grouping file has two columns in which the first column includes samples and the second column are their corresponding groups (e.g. control or disease). The sample grouping file is optional. If not provided, users need to manually select control and disease samples in the GUI (Supplementary Figure S5). The platform supports ID conversion from other gene identifiers to Entrez IDs. The conversion is based on the ID mapping provided by the UniProt database with more than 90 ID types, and 200 annotation packages currently available from Bioconductor (https://bioconductor.org/packages/3.12/data/annotation/).

CPA provides an easy-to-use file manager for users to upload and manage expression data (upload, remove, rename, and download). Users can upload expression data files from their local machine or import them from NCBI GEO. Data importation from GEO is based on the Bioconductor R package GEOquery (47). A dataset can only be imported from GEO if the series matrix (pre-processed gene expression file) is available. Files uploaded and imported by anonymous users will be deleted after 24 hours. Users are encouraged to log onto CPA using a Google account so that they can permanently save data and get access to their analysis sessions across multiple devices.

### Parameter setting for pathway analysis

**Figure 2.**
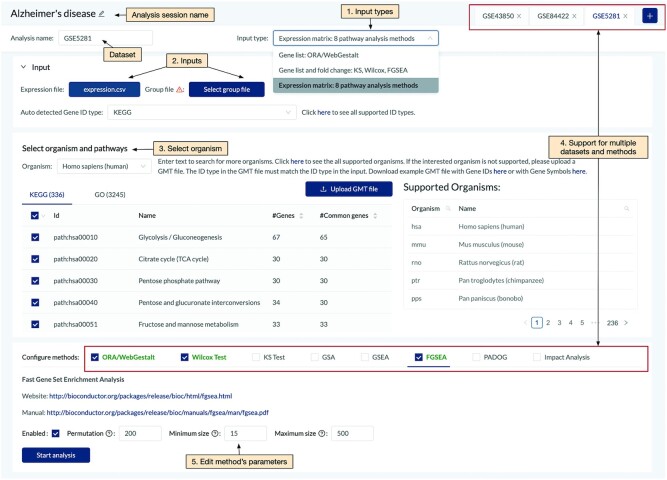
Main components of the pathway analysis page. Users are able to: (1) select input type, (2) select corresponding input with the input type, (3) choose the organism and pathways to be analyzed. The website supports meta-analysis of multiple datasets and multiple methods (4). The website also allows users to change the parameters of individual methods if desired (5).

Figure 2 shows the GUI for pathway analysis, in which users can select one or multiple datasets for an analysis session. For each dataset, users can choose the input type from the drop-down list (see Supplementary Figures S1–S4). When users choose to provide a list of DE genes (gene list), ORA/Webgestalt is available for analysis. When genes and fold changes are chosen, Wilcox-test, KS-test, and FGSEA are available for analysis. When users provide an expression matrix, all of the eight pathway analysis methods are available for analysis: GSEA, GSA, FGSEA, PADOG, Impact Analysis, ORA/WebGestalt, KS-test and Wilcox-test (Supplementary Figure S6). Supplementary Material Section 1 provides brief descriptions for each of the eight methods. Each of them is designed to find different patterns of the data. The purpose of consensus analysis is that users can explore the results of multiple analyses, including results of different datasets as well as of different methods. However, we would also like to note that a particular pathway is identified by multiple methods does not necessarily make it more biologically meaningful.

Currently, CPA supports the analysis of more than 1000 organisms that have KEGG pathways (48) and GO terms (4,49). Users can also upload pathway annotations of other databases in the GMT file format. After choosing data, pathways, and methods, users can start the analysis by simply clicking the ‘Start analysis’ button. Note that classical methods such as ORA, KS or Wilcox test usually take a second to finish the analysis. However, methods such as PADOG or GSEA that involve permutation and bootstrapping usually take several minutes to finish an analysis, especially when analyzing multiple datasets. Analysis sessions are queued and updated in real-time. Results and configurations are saved onto user accounts so that they can switch to any analysis session at any time.

### Analysis and visualization

**Figure 3.**
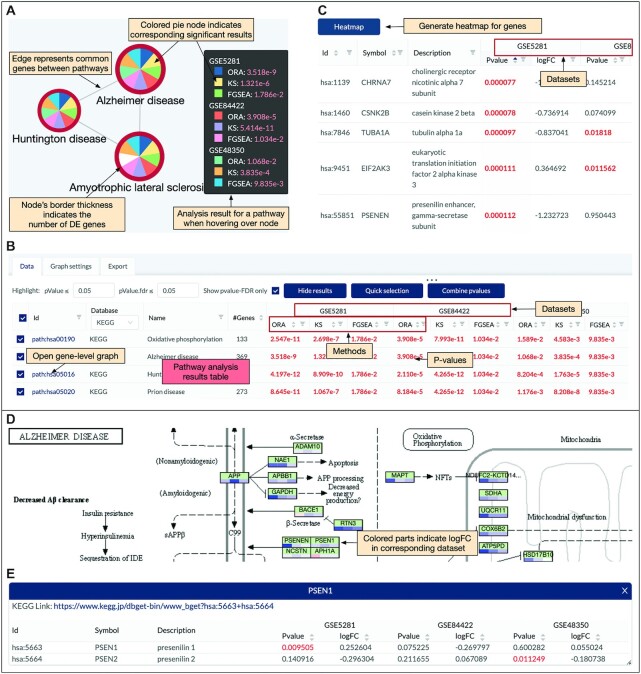
Pathway analysis and visualization using the CPA platform. (**A**) Pathway-pathway connectivity graph where nodes represent pathways and edges represent that there are common genes between pathways. In this example, we analyze three datasets using three methods, making a total of nine analyses. Each node is a pie chart in which individual slices correspond to different analyses. A slice is colored if the corresponding *P*-value is significant. Nodes border’s thickness indicates the number of significantly differentially expressed (DE) genes in the pathway. (**B**) Pathway panel showing the significant pathways and the adjusted *P*-values obtained in each dataset using each analysis method. For example, the *Alzheimer’s disease* pathway is shown on top with significant *P*-values in all of the nine analyses (*P*-values are colored in red when they are significant). This pathway panel is automatically populated, together with the pathway connectivity graph after the analysis is performed. (**C**) Gene panel that appears when users left-click a node in the pathway connectivity graph (in panel A). This panel shows the genes of the pathways and their statistics (*P*-values and log FC) across all datasets. (**D**) Gene network (KEGG pathway) and expression change. This panel appears when users right-click a node in the pathway connectivity graph (in panel A). Nodes in a KEGG pathway graph are divided equally into multiple colored parts representing expression change (up- or down-regulated). (**E**) Gene panel that appears when users right-click on a node of the gene network (in panel D).

Once the analysis is completed, the website displays the pathway-pathway connectivity graph (Figure 3A) in which nodes represent pathways and edges indicate that the connected pathways share a certain number of genes (defined by users). In this pathway graph, the size of a node is proportional with the number of genes in the pathways while the border thickness is proportional with the total number of DE genes. As shown in the figure, each node is divided into multiple slices that represent the results of multiple analyses. For example, an analysis session with three datasets and three methods has a total of nine slices (nine analyses). Users can change the number of nodes displayed by changing the significance threshold (*P*-value) and the number analyses in which the *P*-values are significant. By default, the significance threshold is set to 5% (after adjustment using FDR), and a node appears only if the pathway is significant in at least one analysis. A slice is colored if the pathway has a significant *P*-value in the corresponding analysis. When users hover the mouse over a node, a small window will appear and show the *P*-values of the pathway in all analyses. In Figure 3A, the black window shows the *P*-values of the *Alzheimer’s disease* pathway. All nine *P*-values of this pathway are significant (${\rm FDR}<5\%$) and thus all slices are colored. In contrast, the *Amyotrophic lateral sclerosis* pathway has a white slice because one analysis has a non-significant *P*-value. The graph is highly configurable inasmuch users can easily change the scale and color of all elements to export high-quality figures. Users can also choose to display pathways of only GO, or KEGG, or both (Supplementary Figure S7).

A pathway table that accompanies the pathway graph shows the essential information of each pathway: ID, description, number of genes, and the *P*-values obtained in all analyses (Figure 3B). Using the editable fields and pop-up menus of this table, users can change the significance threshold, filter out pathways, or hide the results of any method or dataset. They can also interactively modify the graph by hiding unwanted pathways or adding pathways of interest. The table can also be used to select pathways with more than a certain number of significant results, or select pathways that are significant in some analyses but not in others. Users can also conduct meta-analysis by combining *P*-values of a pathway across multiple datasets using Fisher’s (50), Stouffer’s (51), addCLT (52), or minP method (53). Note that combining *P*-values obtained from different methods for the same dataset might lead to artificially low meta *P*-values. Therefore, it is recommendable to combine the *P*-values obtained from independent datasets. When combining *P*-values using Fisher’s or Stouffer’s method, any individual *P*-value of zero will result in a combined *P*-value of zero. Therefore, by default, the platform will round the individual *P*-values up to 1e−10 before combining. The meta-analysis results will be added to the pathway table as a column and can also be used to manipulate the pathway graph. The meta-analysis results will be added to the pathway table as a column and can also be used to manipulate the pathway graph.

Besides the pathway table, the platform also creates a gene table (Figure 3C) that appears when users select one or more nodes of the pathway graph. The table shows the genes of the selected pathways, their description, and statistics obtained from all datasets. The table can be modified to show either the intersection or union of all pathways selected. Users can sort the genes, remove unwanted genes, or remove a dataset. The genes and their statistics can be exported. Users can also generate the heatmaps displaying log FC and *P*-values of the genes by just clicking the ‘Heatmap’ button.

The platform also supports pathway visualization. When users right-click on a node of the pathway graph, they can choose to display the KEGG pathway (Figure 3D). In this presentation, each node is a compound. The bar under each node in the pathway is divided into smaller parts that correspond to datasets included in the analysis session. Each part is colored based on its impact direction (i.e., up- or down-regulated). Users can easily find genes that are consistently up- or down-regulated in all datasets and relationships among them. Since each node in a KEGG pathway often includes multiple genes, the color of each part reflects the average FC of all genes in the node. By default, we also combine the *P*-values of all genes of the node to obtain a combined *P*-value. Users can choose to color the node based on this combined *P*-value. Users can also remove any unwanted datasets from the visualization. When users click on a KEGG node, they can see the genes belonging to the node. For example, when clicking on the PSEN1 node on the KEGG pathway, the gene table appears as shown in Figure 3E. This table displays the genes, their description, *P*-value, and log FC in all datasets.

While exploring the analysis results, users can export any graph as raster (.png) or vector (.svg) images. They can also export results obtained from differential analyses, genes information, and *P*-values from pathway analysis as .csv files. Other plots in the pathway analysis page (e.g. sample landscape, volcano plot, etc.) can be export as raster images (.png).

## IMPLEMENTATION

**Figure 4.**
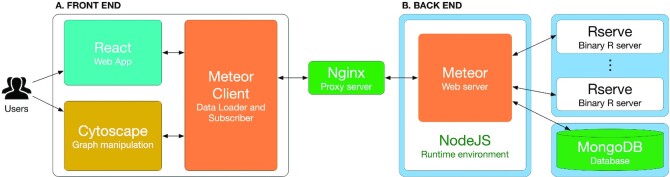
The architecture of the CPA platform. (**A**) Front end that consists of a graphic user interface (using React), graph manipulation module (using Cytoscape) and data handling module (Meteor client). (**B**) Back end that consists of a web server (Meteor web server), runtime environment (NodeJS), R servers (Rserve), and a database (MongoDB). Each backend module is containerized using Docker (blue boxes). The R servers are used to perform pathway analysis while the database is used to store user data and analysis results. User clients (from front end) communicate with back-end servers through the Distributed Data Protocol (Meteor client) and a Nginx web proxy server.

Figure 4 shows the architecture and technologies used to build the CPA platform. We used MeteorJS (https://www.meteor.com/)—a full-stack JavaScript platform for modern web development – as the core web platform to create the web server and to communicate with user clients.

For the front end, we build the graphic user interface using React, which is a JavaScript library (https://reactjs.org/). The website is designed to be user-friendly with three main pages: pathway analysis, pathway visualization, and data management. In the pathway analysis page, users can upload and choose datasets, select methods, and observe gene-level statistics. Gene-level plots are generated using the Plotly JavaScript graphing library (https://plotly.com/javascript/). In the visualization page, we implement the interactive network visualization using CytoscapeJS, which is a graph theory library (https://js.cytoscape.org/). Gene heatmaps are plotted using D3js (https://d3js.org/). In the data management page, we build the file manager using OpusCapita React File Manager (https://www.npmjs.com/package/@opuscapita/react-filemanager). Data exchange between user clients and back-end servers is accomplished using the Distributed Data Protocol (Meteor client) and a Nginx web proxy server (https://www.nginx.com).

For the back end, we build the webserver using Meteor and NodeJS (https://nodejs.org). NodeJS is a JavaScript runtime environment built on Chrome’s V8 JavaScript engine that allows JavaScript code to run outside the browser environments. Input files for analysis are stored on the server’s storage for fast access. Other data including user information, analysis sessions, analysis configuration, and results are saved in a MongoDB database (https://www.mongodb.com). Once the requests for performing pathway analysis are received from clients and saved by the web server, they are passed onto R servers created by Rserve (https://www.rforge.net/Rserve/) to perform pathway analyses. Multiple Rserve instances can be created to perform multiple analyses concurrently. All software and packages in the back end run in containerized environments using Docker (https://www.docker.com/).

## DATA SOURCE

CPA supports the analysis of more than 1000 organisms using KEGG (48) and GO terms (4). At the time of writing this article, the version of KEGG is 97.0 (released 1 January 2021) and of GO terms is 1.16 (released 16 February 2021). The automatic ID conversion in the CPA platform is based on the ID mapping from the UniProt database (current version: 2021_02) and more than 200 annotation packages from Bioconductor (version 3.12, released 28 October 2020). ID mappings and databases will be updated twice a year (January and July).

## RESULTS

To show how the CPA platform can be used for pathway analysis, we have created an example analysis session and include it in our tutorial page. In this example session, we analyze three Alzheimer’s datasets: GSE5281 (54), GSE84422 (55), and GSE48350 (56). The three datasets consist of a total of 66 control and 57 disease samples (Table 1). We choose the Alzheimer’s datasets because there is a target pathway in KEGG, Alzheimer’s disease, that describes the known mechanisms and biological processes involved in this disease. It is also well-known that the pathways Parkinson’s disease, Huntington’s disease, and Pathways of neurodegeneration - multiple diseases share many genes and mechanisms with Alzheimer’s disease (57–60). Therefore, we expect to identify all these neurological disorder pathways as statistically significant.

**Table 1.** Alzheimer’s datasets used in our data analysis. The first two columns show the accession ID and tissue while the last three columns show the number of controls, number of diseases and assaying platforms, respectively

Dataset	Tissue	C	D	Platform
GSE5281	Entorhinal cortex	13	10	HG-U133+ 2.0
GSE84422	Sup. Tem. gyrus	14	22	HG-U133A
GSE48350	Entorhinal cortex	39	15	HG-U133A

**Figure 5.**
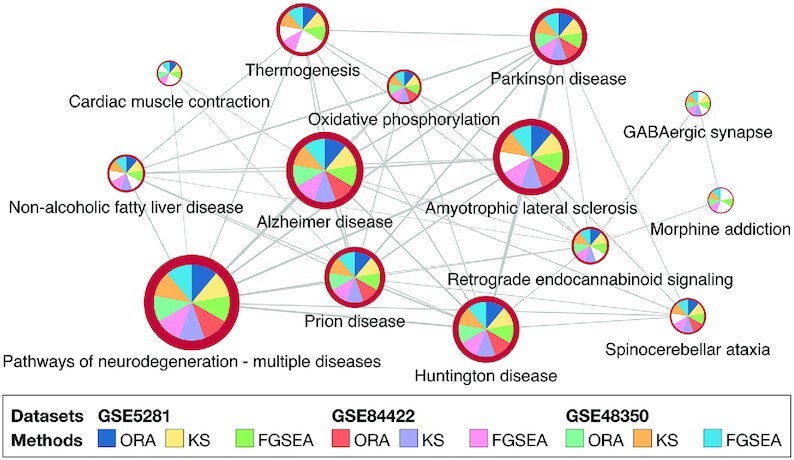
The connected module of pathways that are significantly impacted in Alzheimer’s datasets GSE5281, GSE84422 and GSE48350. Each dataset is analyzed using three pathway methods, ORA, KS-test, and FGSEA. Only pathways that are significantly impacted in at least 5 analyses (out of 9) are shown.

In this analysis, we include a total of 335 KEGG pathways and 2508 GO terms. In the global pathway–pathway connectivity graph, we have a total of 2843 nodes—one node per KEGG pathway or GO term. Each dataset is analyzed with three methods, ORA, KS-test, and FGSEA, using default parameters. For each analysis, we adjust the *P*-values using Benjamini–Hochberg’s False Discovery Rate (FDR) (61). The significance threshold is set to ${\rm FDR}<5\%$. Figure 5 shows the subnetwork obtained with the significant nodes. Nodes in the module are selected so that each pathway is significantly impacted in at least five analyses (out of nine analyses).

The five pathways related to neurodegenerative diseases, Pathways of neurodegeneration - multiple diseases, Alzheimer’s disease, Huntington’s disease, Parkinson’s disease, and Prion disease, are consistently identified as significant in all of the nine analyses. The Amyotrophic lateral sclerosis pathway is significant in eight out of nine.

Table 2 shows the FDR-corrected *P*-values of the 14 pathways. The first column shows the pathway name while the next nine columns show the *P*-values obtained from the nine analyses. As the web interface also allows us to combine the *P*-values obtained for a pathway across multiple datasets, we use the addCLT method (52) to combine the *P*-values for each method. The meta-analysis results are presented in the three last columns in Table 2. The meta-analysis, as well as the results obtained from individual analyses, clearly shows that pathways related to neurodegenerative diseases are significantly impacted regardless of datasets and methods.

**Table 2.** FDR-corrected *P*-values of 14 pathways that are significantly impacted in three Alzheimer’s datasets (GSE5281, GSE84422, and GSE48350). Each dataset is analyzed by three methods (ORA, KS-test, and FGSEA), resulted in 9 analyses (columns 3–11). The last three columns show the meta-analysis results using the addCLT method. The results indicate that these pathways are consistently identified as significant across all analyses

		GSE5281			GSE84422			GSE48350			Meta-analysis		
#	Pathway name	ORA	KS	FGSEA	ORA	KS	FGSEA	ORA	KS	FGSEA	ORA	KS	FGSEA
1	Alzheimer disease	4e-09	1e-06	2e-02	4e-05	5e-11	1e-02	1e-02	4e-04	1e-02	4e-07	9e-13	2e-06
2	Huntington disease	4e-12	9e-10	2e-02	2e-05	4e-12	1e-02	8e-04	2e-05	1e-02	6e-11	3e-17	2e-06
3	Parkinson disease	0	4e-13	2e-02	4e-07	0	1e-02	2e-03	1e-04	1e-02	6e-10	2e-14	2e-06
4	Prion disease	9e-11	1e-07	2e-02	8e-05	4e-12	1e-02	1e-03	8e-08	1e-02	2e-10	8e-24	2e-06
5	Pathways of neurodegeneration	1e-11	2e-08	2e-02	4e-06	4e-10	1e-02	2e-03	7e-06	1e-02	3e-10	1e-18	2e-06
6	Oxidative phosphorylation	3e-11	3e-07	2e-02	4e-05	8e-11	1e-02	2e-02	5e-03	1e-02	1e-06	1e-08	2e-06
7	Cardiac muscle contraction	2e-02	1e-02	2e-02	6e-01	3e-01	1e-02	4e-02	9e-02	1e-02	3e-02	1e-02	2e-06
8	Thermogenesis	4e-05	1e-02	2e-02	3e-01	5e-02	1e-02	3e-01	9e-03	1e-02	4e-02	6e-06	2e-06
9	Retrograde endocannabinoid s.	8e-06	1e-03	2e-02	4e-01	7e-04	1e-02	3e-04	3e-07	1e-02	4e-03	3e-11	2e-06
10	Amyotrophic lateral sclerosis	2e-09	2e-06	2e-02	2e-07	1e-10	1e-02	1e-01	2e-03	1e-02	3e-03	6e-10	2e-06
11	GABAergic synapse	8e-02	4e-02	3e-02	9e-02	4e-02	1e-02	4e-03	3e-03	1e-02	7e-05	7e-06	4e-06
12	Spinocerebellar ataxia	4e-03	1e-02	2e-02	3e-03	1e-05	1e-02	8e-02	3e-03	1e-02	3e-04	5e-08	2e-06
13	Non-alcoholic fatty liver d.	7e-06	6e-03	2e-02	3e-01	1e-04	1e-02	2e-01	1e-02	5e-02	2e-02	3e-07	8e-05
14	Morphine addiction	4e-01	7e-01	3e-02	1E+00	6e-01	4e-02	5e-03	9e-04	1e-02	3e-01	8e-01	3e-05

**Figure 6.**
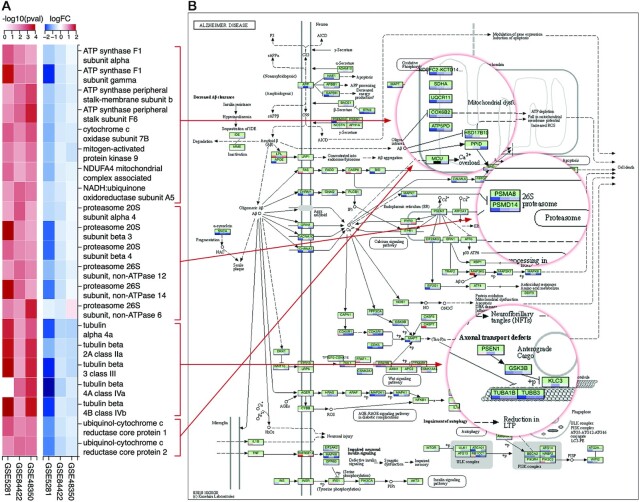
Differential analysis of genes that belong to five neurodegenerative pathways: Pathways of neurodegeneration - multiple diseases, Alzheimer’s disease, Huntington’s disease, Parkinson’s disease, and Prion disease. (**A**) Heatmaps of *P*-values and log FC of genes that are differentially expressed (DE) in all of the three Alzheimer’s datasets (GSE5281, GSE84422, and GSE48350). (**B**) Mapping of DE genes to mitochondria, proteasome, and microtubule components of the *Alzheimer’s disease* pathway.

Using the website, we also perform a gene-level analysis to identify genes that can potentially play an important role in the dysregulation of the five neurodegenerative pathways. For that purpose, we intersect the genes that: (i) belong to all of the five pathways and (ii) are differentially expressed in all three datasets (${\rm FDR}<5\%$). Figure 6A shows the heatmaps of the resulting 21 DE genes. Most of these genes belong to the components related to mitochondria, proteasome, and microtubule in all five pathways. Figure 6B shows the direct mapping of these genes to those components of the Alzheimer’s disease pathway.

## CONCLUSIONS

In this article we describe a new web-based platform named CPA that allows researchers to: (i) analyze gene/protein expression data using eight popular methods (GSEA, GSA, FGSEA, PADOG, Impact Analysis, Webgestalt, KS-test, Wilcox-test), (ii) perform meta-analysis of multiple datasets, (iii) combine methods and datasets to find consensus results and (iv) interactively explore significantly impacted pathways across multiple analyses, and browse relationships between pathways and genes. Our main objective is to help life scientists who are trying to understand the underlying biological mechanisms when comparing two phenotypes. The platform is user-friendly with rich features to explore and visualize pathway analysis results. More importantly, it allows users to see the differences, as well as the consensus results across many methods and experiments. At the same time, we also aim at helping bioinformaticians who are developing new pathway analysis methods.

## DATA AVAILABILITY

The Consensus Pathway Analysis (CPA) platform is available at http://cpa.tinnguyen-lab.com. This web application is free and open to all users. The platform also includes a tutorial page with step-by-step instruction and example analyses.

## Supplementary Material

gkab421_Supplemental_File
