# Supplementary material for: CPA: a web-based platform for consensus pathway analysis and interactive visualization
Source: Nucleic Acids Res. 2021 May 25;49(W1):W114–24. doi: 10.1093/nar/gkab421 (PMC8262702; doi:10.1093/nar/gkab421)
Supplement: gkab421_Supplemental_File [file gkab421_supplemental_file.pdf]

CPA: A web-based platform for Consensus Pathway Analysis and interactive visualization

Supplementary Material

Hung Nguyen [1], Duc Tran [1], Jonathan M. Galazka [2], Sylvain V. Costes [2], Afshin Beheshti [3], Juli Petereit [4], Sorin Draghici [5], Tin Nguyen [1*]

[1]University of Nevada Reno, Department of Computer Science and Engineering, Reno, NV 89557, USA,
[2]NASA Ames Research Center, Space Biosciences Division, Moffett Field, CA 94035, USA,
[3]KBR, NASA Ames Research Center, Space Biosciences Division, Moffett Field, CA 94035, USA,
[4]University of Nevada Reno, Nevada Bioinformatics Center, Reno, NV 89557, USA and
[5]Wayne State University, Department of Computer Science, Detroit, MI 48202, USA

Contents

1 Pathway Analysis Methods **3**
1.1 Over-representation analysis (ORA) 3
1.2 Gene set enrichment analysis (GSEA) 3
1.3 Gene set analysis (GSA) 3
1.4 Fast Gene Set Enrichment Analysis (FGSEA) 4
1.5 Pathway analysis with down-weighting overlapping genes (PADOG) 4
1.6 Kolmogorov-Smirnov (KS) test 4
1.7 Wilcoxon test 4
1.8 Impact analysis 5

2 CPA Interface **5**

1 Pathway Analysis Methods

Pathway analysis methods can be categorized into three classes. The earliest approaches use Over-Representation Analysis (ORA) [1–6] that identify the pathways in which the DE genes are over- or under-represented. Functional Class Scoring (FCS) approaches [7–11] have been developed to address some of the issues raised by ORA approaches. The main improvement of FCS is based on the observation that small but coordinated changes in expression of functionally related genes can have significant impact on pathways. However, both ORA and FCS still ignore the direction and type of the signals between genes, the positions and roles of the genes on each pathway, as well as all the other information captured by the topology of the pathway. Topology-based (TB) approaches [12–19] which fully exploit all the knowledge about how gene interact as described by pathways, have been developed more recently.

Our website implements eight pathway analysis methods. They can be categorized into the above three categories: i) Over-representation analysis; ii) Functional Class Scoring: Gene set enrichment analysis (GSEA), Gene set analysis (GSA), Fast Gene Set Enrichment Analysis (FGSEA), Pathway analysis with down-weighting overlapping genes (PADOG), Kolmogorov-Smirnov (KS) test, and Wilcoxon (Wilcox) test; and iii) Topology-based: Impact Analysis. The detail about these methods are described in the following subsections.

1.1 Over-representation analysis (ORA)

Over-representation analysis (ORA) [20] is a method that tests whether the number of differentially expressed genes are over-represented in a gene set. The null hypothesis is that genes in the uploaded list of differentially expressed (DE) genes are sampled from the same general population as genes from the reference set, i.e. the probability of observing a DE gene from a particular gene set GS is the same as observing at other genes in the reference list. The alternative hypothesis is that the differentially expressed genes are over- or under-represented in the gene set. ORA uses hypergeometric test to calculate the p-value that represents how likely one can observes that many DE gene in the gene set just by chance.

1.2 Gene set enrichment analysis (GSEA)

The null hypothesis of GSEA [7, 21] is that "*the rank ordering of genes in a given comparison is random with regard to the diagnostic categorization of the samples*". The alternative hypothesis is that "*the rank ordering of the pathway members is associated with the specific diagnostic criteria used to categorize the groups of affected individuals*" [21].

Denote N as the total number of genes, GS_i as the ith gene set, n_i as the number of genes in the $i^{th} geneset$, $(z_1, z_2, \dots, z_{n_i})$ as the t-statistic of genes in the ith gene set. For gene set GS_i, GSEA computes a score $S(GS_i)$ which essentially equals to a signed version of the Kolmogorov-Smirnov statistic between the values z_j $(j \in GS_i)$ and their complement. The samples then are permuted many times to build the empirical null distribution of the score for each gene set. The significance of the ith gene set is determined by the fraction of the distribution that is more extreme than the observed $S(GS_i)$.

1.3 Gene set analysis (GSA)

GSA [8] differs from GSEA mainly in two ways: the summary statistic and the re-standardization of gene set scores based on row randomization. First, the score of the gene set is the maxmean statistic:

$$S_{max}(GS_i) = \max(\frac{\sum z_j^{(+)}}{n_i}, \frac{\sum z_j^{(-)}}{n_i}) \tag{1}$$

where the (+) and (-) signs identify the positive and negative t-scores, respectively, and n_i is the number of genes in the gene set. Second, GSA re-standardizes the gene set scores by taking into account scores from sets formed by random selection of genes. GSA then permutes the samples to compute the significance of the standardized gene set scores.

1.4 Fast Gene Set Enrichment Analysis (FGSEA)

Fast Gene Set Enrichment Analysis (FGSEA) [22, 23] has the same null and alternative hypotheses as GSEA. FGSEA differs from GSEA in the idea of reusing sampling for different query gene-sets. Instead of generating n independent random gene sets for each of M input pathways (total of $n * M$), FGSEA will generate only n random gene sets of size K. K is equal to the size of the biggest pathway. Let g_i be an ith random gene set of size K. From that gene set we can generate gene sets for all the query pathways P_j by using its prefix: $g_{i,j} = g_i[1..K_j]$, where K_j is the size of pathway P_j. The next step is to calculate the enrichment scores for all gene sets $g_{i,j}$. Instead of calculating Enrichment Scores separately for each gene set, FGSEA will calculate simultaneously scores for all $g_{i,j}$ for a fixed i.

Another improvement of FGSEA is that given a gene set sample g_i of the size K, the Enrichment Score values for all the prefixes $g_{i,1..j}$ can be calculated in an efficient manner using a square root heuristic. Briefly, a variant of an enrichment curve is considered: the genes are enumerated starting from the most up-regulated to the most down-regulated, with the curve going to the right if the gene is not present in the pathway, and the curve goes upward if the gene is present in the pathway.

With these two improvements, the time complexity of the calculating P-values for the set of M pathways is $O(n(K\sqrt{K}+M))$, which gives around $O(\sqrt{K}\log(K))$ speed up compared to a naive approach. This allows FGSEA to perform analysis with much higher number of permutation, which leads to the ability to estimate lower value of p.

1.5 Pathway analysis with down-weighting overlapping genes (PADOG)

The null hypothesis of Pathway analysis with down-weighting overlapping genes (PADOG) [10, 24] is that the mean of the (weighted) absolute differences between the phenotypes for the genes on a given pathway is zero. The alternative hypothesis is that this mean is different from zero. An alternative formulation is that the null hypothesis states that no gene on the pathway is a DEG, with the alternative stating that there is at least a gene that is a differentially expressed gene (DEG) on the given pathway. This formulation of the null hypothesis belongs to the self-contained category of null hypotheses according to [25] and in the second type of null hypotheses according to [26]. The statistic for the gene set GS_i is as follows:

$$S(GS_i) = \frac{1}{n_i}\sum_{j=1}^{n_i} |\mathcal{T}(g_j)| \cdot w(g_j) \tag{2}$$

where n_i is the number of genes in the gene set, g_j ($j \in [1..n_i]$) are the genes in the gene sets, $\mathcal{T}(g_j)$ is the moderate t-score of the gene g_j, $w(g_j)$ is the weight for gene g_j. A gene is weighted less if it appears in more gene sets. The score is then standardized based on row randomization. PADOG then permutes the samples to compute the significance of the standardized gene set scores.

1.6 Kolmogorov-Smirnov (KS) test

Kolmogorov-Smirnov (KS) [27] test compares two empirical distributions to determine whether they differ significantly. It is a non-parametric test that does not make any assumptions about the distributions of the given data sets. In the context of pathway analysis, the two empirical distributions are the scores of the DE genes inside (denoted as DE-hit) and outside (denoted as DE-miss) a pathway. The null hypothesis here is that there is no association between DE genes and the given pathway, and therefore, there is no significant difference between the two empirical distributions of DE-hit and DE-miss. The alternative hypothesis is that there is a difference between the two empirical distribution of DE-hit and DE-miss.

1.7 Wilcoxon test

Wilcoxon (Wilcox) test [28] is a non-parametric statistical test generally used to determine whether or not there is a significant difference in the medians of two given populations. In the context of pathway

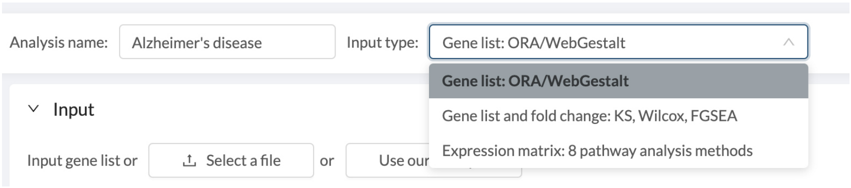

Figure S1: Input types in the CPA websites. Supported input include: 1) a list of differentially expressed genes, 2) a list of genes and their fold changes, and 3) a full expression matrix.

analysis, Wilcox test can be used to compare the ranks or p values (derived from a statistical test, such as a t-test) of the DE genes inside and outside a pathway. Wilcox takes the list of DE genes, their fold changes, and a list of genes of a given pathway as input. The null hypothesis here is there is no significant difference between the statistics medians of the DE genes inside and outside a pathway. The alternative hypothesis is that the statistics median of DE genes inside a pathway is different from that of DE genes outside that pathway.

1.8 Impact analysis

Impact analysis [13] performs two simultaneous tests: one is focused on the number of differentially expressed genes (DEGs) that fall on a given pathway, while the other one focuses on the amount of perturbation accumulation observed on a pathway. The first p-value aims to characterize the enrichment of the pathway in DEGs. The null hypothesis for this test is that the proportion of DEGs on the pathway is less than or equal to the overall proportion of DEGs. The alternative hypothesis is that the proportion of DEGs on the pathway is higher than the overall proportion of DEGs (one-tail test for enrichment). The second test is concerned with the location, magnitude and sign of DEGs on the given pathway. The null hypothesis is that the DEGs appear at random positions in the pathway and that they have random differential expression. The alternative hypothesis is that these DEGs are not randomly distributed on the pathway and their direction of change is somewhat coherent with the direction of change of upstream genes and the previously known type of relations between genes. The null distribution of the overall pathway perturbation accumulation is obtained by randomly permuting the DEG at different locations in the pathway graph. The two types of evidences captured in the form of p-values (enrichment and topological) are then combined using Fisher's method.

2 CPA Interface

The CPA website supports three different types of input: a gene list, a gene list and their fold changes, or a gene expression matrix (Figure S1). The GUI interfaces for different input types are shown in Figures S2-S4. Figure S5 shows the GUI interface of choosing samples for each group (control vs. condition). Figure S6 shows the parameter setting for ORA. Figure S7 show the drop-down box for selecting the database for visualization.

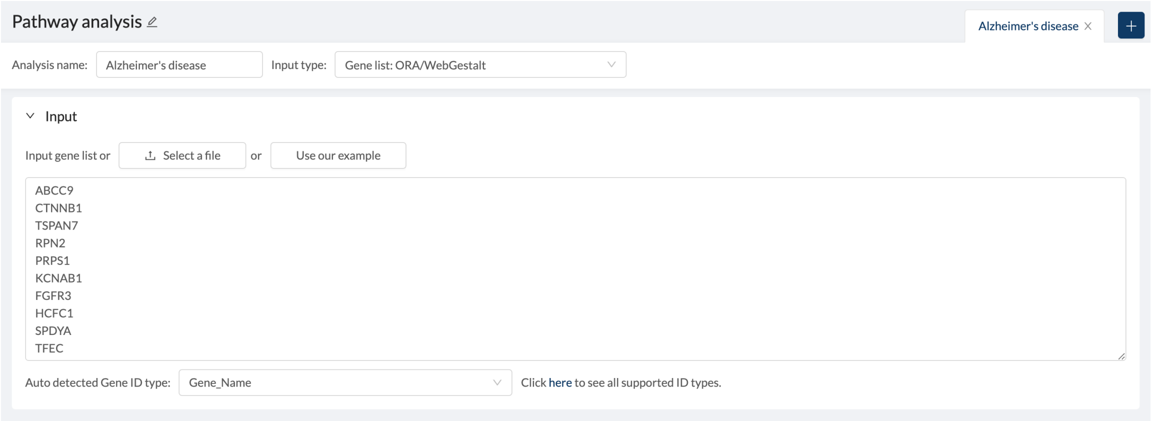

Figure S2: The interface that allows users to input a list of differentially expressed genes.

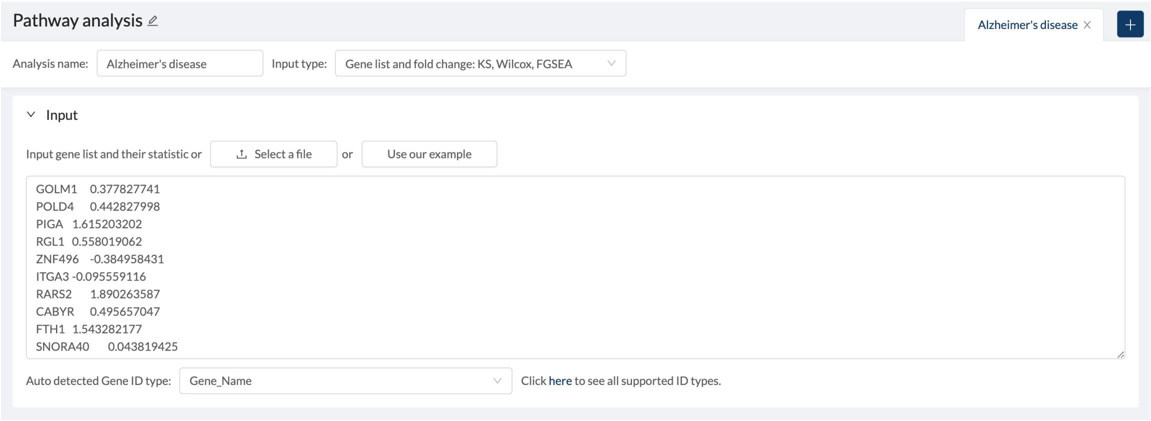

Figure S3: The interface that allows users to input a list of genes and their fold changes.

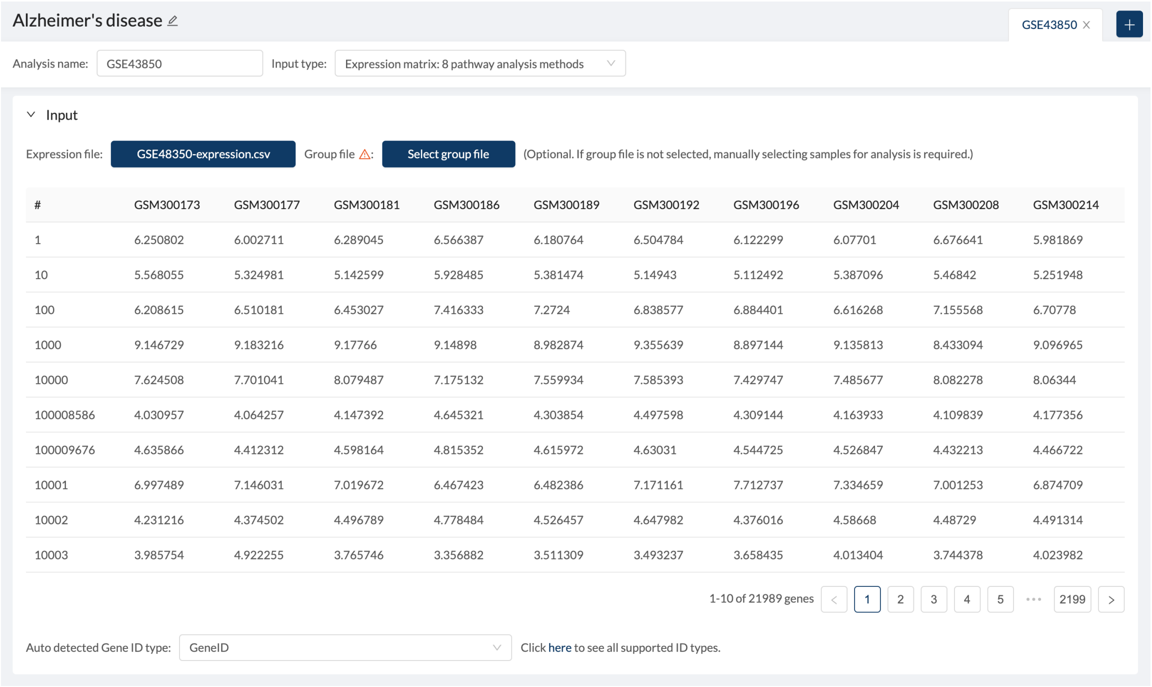

Figure S4: The interface that allows users to input a gene expression matrix.

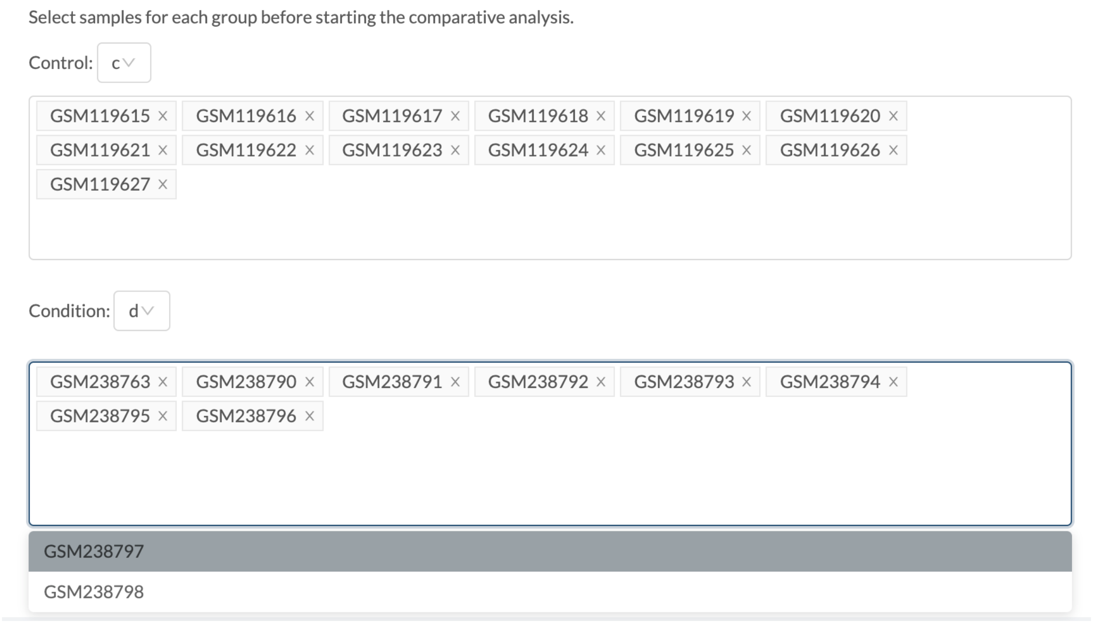

Figure S5: Interface for choosing samples of each sample group (controls vs. condition).

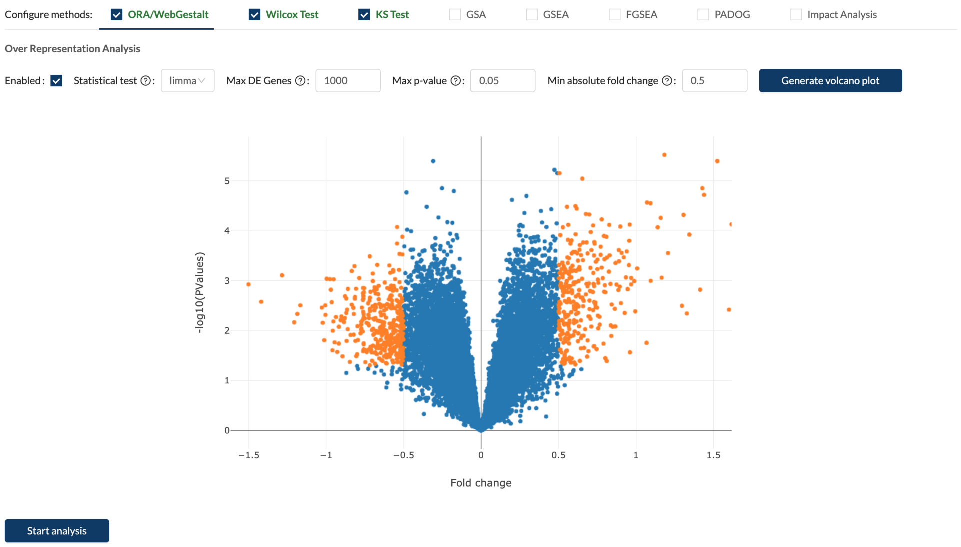

Figure S6: Gene filtering options for ORA method.

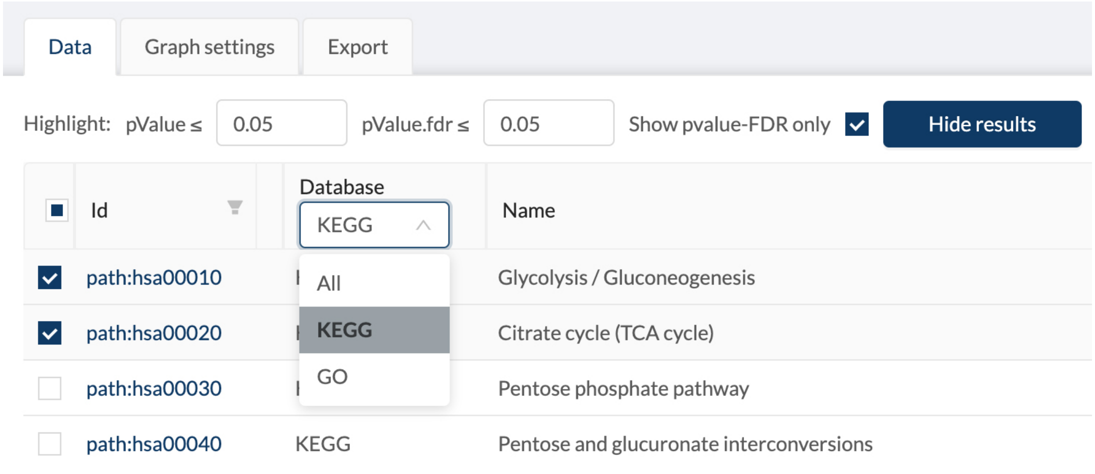

Figure S7: Database seletion for pathway visualization. If a database is selected, the graph will only show pathways that belong to that database.

References

[1] Khatri, P., Drăghici, S., Ostermeier, G. C., and Krawetz, S. A. (2002) Profiling gene expression using Onto-Express. *Genomics,* **79**(2), 266–270.

[2] Hosack, D. A., Dennis Jr., G., Sherman, B. T., Lane, H. C., and Lempicki., R. A. (2003) Identifying Biological Themes within Lists of Genes with EASE.. *Genome Biology,* **4**(6), P4.

[3] Al-Shahrour, F., Diaz-Uriarte, R., and Dopazo, J. (2004) FatiGO: a web tool for finding significant associations of Gene Ontology terms with groups of genes. *Bioinformatics,* **20**(4), 578–580.

[4] Beißbarth, T. and Speed, T. P. (June, 2004) GOstat: find statistically overrepresented Gene Ontologies within a group of genes.. *Bioinformatics,* **20**, 1464–1465.

[5] Huang, D. W., Sherman, B. T., and Lempicki, R. A. (2009) Systematic and integrative analysis of large gene lists using DAVID bioinformatics resources. *Nature Protocols,* **4**(1), 44.

[6] Wang, J., Duncan, D., Shi, Z., and Zhang, B. (2013) WEB-based GEne SeT AnaLysis Toolkit (WebGestalt): update 2013. *Nucleic Acids Research,* **41**(W1), W77–W83.

[7] Subramanian, A., Tamayo, P., Mootha, V. K., Mukherjee, S., Ebert, B. L., Gillette, M. A., Paulovich, A., Pomeroy, S. L., Golub, T. R., Lander, E. S., and Mesirov, J. P. (2005) Gene set enrichment analysis: a knowledge-based approach for interpreting genome-wide expression profiles. *Proceeding of The National Academy of Sciences,* **102**(43), 15545–15550.

[8] Efron, B. and Tibshirani, R. (2007) On testing the significance of sets of genes. *The Annals of Applied Statistics,* **1**(1), 107–129.

[9] Jiang, Z. and Gentleman, R. (2007) Extensions to gene set enrichment. *Bioinformatics,* **23**(3), 306–313.

[10] Tarca, A. L., Drăghici, S., Bhatti, G., and Romero, R. (2012) Down-weighting overlapping genes improves gene set analysis. *BMC Bioinformatics,* **13**(1), 136.

[11] Kong, S. W., Pu, W. T., and Park, P. J. (2006) A multivariate approach for integrating genome-wide expression data and biological knowledge. *Bioinformatics,* **22**(19), 2373–2380.

[12] Rahnenführer, J., Domingues, F. S., Maydt, J., and Lengauer, T. (2004) Calculating the Statistical Significance of Changes in Pathway Activity From Gene Expression Data. *Statistical Applications in Genetics and Molecular Biology,* **3**(1).

[13] Draghici, S., Khatri, P., Tarca, A. L., Amin, K., Done, A., Voichiţa, C., Georgescu, C., and Romero, R. (2007) A systems biology approach for pathway level analysis. *Genome Research,* **17**(10), 1537–1545.

[14] Tarca, A. L., Drăghici, S., Khatri, P., Hassan, S. S., Mittal, P., Kim, J.-s., Kim, C. J., Kusanovic, J. P., and Romero, R. (2009) A novel signaling pathway impact analysis. *Bioinformatics,* **25**(1), 75–82.

[15] Shojaie, A. and Michailidis, G. (2009) Analysis of Gene Sets Based on the Underlying Regulatory Network. *Journal of Computational Biology,* **16**(3), 407–426.

[16] Glaab, E., Baudot, A., Krasnogor, N., and Valencia, A. (2010) TopoGSA: network topological gene set analysis. *Bioinformatics,* **26**(9), 1271–1272.

[17] Greenblum, S., Efroni, S., Schaefer, C., and Buetow, K. (2011) The PathOlogist: an automated tool for pathway-centric analysis. *BMC Bioinformatics,* **12**(1), 133.

[18] Gu, Z., Liu, J., Cao, K., Zhang, J., and Wang, J. (2012) Centrality-based pathway enrichment: a systematic approach for finding significant pathways dominated by key genes. *BMC Systems Biology,* **6**(1), 56.

[19] Gu, Z. and Wang, J. (2013) CePa: an R package for finding significant pathways weighted by multiple network centralities. *Bioinformatics,* **29**(5), 658–660.

[20] Drăghici, S., Khatri, P., Martins, R. P., Ostermeier, G. C., and Krawetz, S. A. (2003) Global functional profiling of gene expression. *Genomics,* **81**(2), 98–104.

[21] Mootha, V. K., Lindgren, C. M., Eriksson, K.-F., Subramanian, A., Sihag, S., Lehar, J., Puigserver, P., Carlsson, E., Ridderstråle, M., Laurila, E., Houstis, N., Daly, M. J., Patterson, N., Mesirov, J. P., Golub, T. R., Tamayo, P., Spiegelman, B., Lander, E. S., Hirschhorn, J. N., Altshuler, D., and Groop, L. C. (2003) PGC-11α-responsive genes involved in oxidative phosphorylation are coordinately downregulated in human diabetes. *Nature Genetics,* **34**(3), 267–273.

[22] Korotkevich, G., Sukhov, V., Budin, N., Shpak, B., Artyomov, M. N., and Sergushichev, A. (2021) Fast gene set enrichment analysis. *BioRxiv,* p. 060012.

[23] Sergushichev, A. A. (2016) An algorithm for fast preranked gene set enrichment analysis using cumulative statistic calculation. *BioRxiv,* p. 060012.

[24] Tarca, A. L., Bhatti, G., and Romero, R. (2013) A comparison of gene set analysis methods in terms of sensitivity, prioritization and specificity. *PloS One,* **8**(11), e79217.

[25] Emmert-Streib, F. and V. Glazko, G. (2011) Pathway Analysis of Expression Data: Deciphering Functional Building Blocks of Complex Diseases. *PLoS Computational Biology,* **7**(5), e1002053.

[26] Tian, L., Greenberg, S. A., Kong, S. W., Altschuler, J., Kohane, I. S., and Park, P. J. (2005) Discovering statistically significant pathways in expression profiling studies. *Proceeding of The National Academy of Sciences,* **102**(38), 13544–13549.

[27] Stuart, A., Arnold, S., Ord, J. K., O'Hagan, A., and Forster, J. (1994) Kendall's advanced theory of statistics, Vol. 1, Wiley, London 6th edition.

[28] Wilcoxon, F. (1945) Individual comparisons by ranking methods. *Biometrics,* **1**(6), 80–83.
